# Genome-scale identification and comparative analysis of transcription factors in thermophilic cyanobacteria

**DOI:** 10.1186/s12864-024-09969-7

**Published:** 2024-01-09

**Authors:** Jie Tang, Zhe Hu, Jing Zhang, Maurycy Daroch

**Affiliations:** 1 School of Pharmacy and Bioengineering, Chengdu University, Chengdu, 610106 China; 2 Food Safety Detection Key Laboratory of Sichuan, Technical Center of Chengdu Customs, Chengdu, 610041 China; 3 School of Environment and Energy, Peking University Shenzhen Graduate School, Shenzhen, 518055 China

**Keywords:** Thermophilic cyanobacterium, Transcription factor, Comparative genomics

## Abstract

**Background:**

The transcription factors (TFs) in thermophilic cyanobacteria might represent a uniquely evolved gene repertoire in light of the strong selective pressure caused by hostile habitats. Understanding the molecular composition of the TF genes in thermophilic cyanobacteria will facilitate further studies regarding verifying their exact biochemical functions and genetic engineering. However, limited information is available on the TFs of thermophilic cyanobacteria. Herein, a thorough investigation and comparative analysis were performed to gain insights into the molecular composition of the TFs in 22 thermophilic cyanobacteria.

**Results:**

The results suggested a fascinating diversity of the TFs among these thermophiles. The abundance and type of TF genes were diversified in these genomes. The identified TFs are speculated to play various roles in biological regulations. Further comparative and evolutionary genomic analyses revealed that HGT may be associated with the genomic plasticity of TF genes in *Thermostichus* and *Thermosynechococcus* strains. Comparative analyses also indicated different pattern of TF composition between thermophiles and corresponding mesophilic reference cyanobacteria. Moreover, the identified unique TFs of thermophiles are putatively involved in various biological regulations, mainly as responses to ambient changes, may facilitating the thermophiles to survive in hot springs.

**Conclusion:**

The findings herein shed light on the TFs of thermophilic cyanobacteria and fundamental knowledge for further research regarding thermophilic cyanobacteria with a broad potential for transcription regulations in responses to environmental fluctuations.

**Supplementary Information:**

The online version contains supplementary material available at 10.1186/s12864-024-09969-7.

## Background

Thermophilic cyanobacteria are oxygen-evolving photosynthetic prokaryotes that show a ubiquitous distribution in diverse thermal environments around the world [1–3]. The importance of thermophilic cyanobacteria has been demonstrated in numerous studies mainly due to their contribution to a large part of geothermal ecosystems’ biomass and productivity [4]. Moreover, thermophilic cyanobacteria have been explored for various applications concerning agriculture, pharmaceutics, nutraceutical, and biofuel [5], and have been manipulated to improve the production capability of cyanofactories by various biotechnological approaches, such as metabolic engineering [6]. Nevertheless, bottlenecks are widespread in the application of cyanobacteria in biotechnology [7]. Therefore, to fully explore the industrial potential of cyanobacteria requires thorough studies on each biological block of thermophilic cyanobacteria.

Genome expression modulation is crucial for every living organism and is the underlying mechanisms of development, morphology and physiology [8]. Gene expression is one level of this modulation, in which transcription factors (TFs) are one of the key players [9]. TFs usually are structurally composed of a DNA binding domain (DBD), an oligomerization domain responsible for interaction with other TFs, and a transcription regulation domain controlling gene expression [10]. The sequences of most TFs possess only one type of DBD in one or multiple copies, while several DBD types are also present in some TFs [11]. The TF proteins affect the expression of multiple target genes by binding to specific DNA motifs in their promoter regions.

As the increasing number of prokaryotes with complete genome sequences, *in silico* studies have been extensively performed to identify putative TFs in prokaryotic genomes [10, 12]. More importantly, taxonomically diverse data facilitate comparative analyses between different species or lineages, further providing insights into taxonomic characteristics of the TF complement of different organisms. In the past decade, many cyanobacterial genomes from thermal environments have been elucidated [13, 14]. However, the TF genes in thermophilic cyanobacteria are rarely characterized.

The utilization of TFs has been widely applied in the construction of synthetic genetic networks for cyanobacteria [7, 15]. Cyanobacterial strains with engineered transcription machinery might provide solutions for construction of highly efficient production platforms for biotechnical applications in the future. Furthermore, thermophilic cyanobacteria might evolve into a unique repertoire of TF genes in light of the strong selective pressure caused by hostile habitats. Understanding the molecular composition of the TF genes in thermophilic cyanobacteria will be helpful as a prerequisite for verifying their exact biochemical functions and for further genetic engineering.

Herein, we carried out genome-wide identification and comparison of the TF repertoire of thermophilic cyanobacteria. The TF composition were thoroughly analyzed and compared. Special focus was given to the genus and strain-specific TFs between these thermophilic cyanobacteria and corresponding mesophilic cyanobacterium, and their putative functions was discussed.

## Materials and methods

### Source dataset of thermophilic cyanobacteria

A total of 22 thermophilic cyanobacteria were compiled into the genome dataset. These thermophilic cyanobacteria were previously verified by the literatures [16–18]. Briefly, the 22 thermophiles were taxonomically assigned to six families, including Leptolyngbyaceae: *Leptodesmis sichuanensis* A121 [19] and *Leptothermofonsia sichuanensis* E412 [20]; Oculatellaceae: *Leptolyngbya* sp. JSC-1 [21], *Thermocoleostomius sinensis* A174 [22], *Thermoleptolyngbya* sp. O-77 [23] and *T. sichuanensis* A183 [24]; Gloeomargaritaceae: *Synechococcus* sp. C9 [25]; Thermostichaceae: *Thermostichus* sp. 60AY4M2, 63AY4M2, 65AY6A5, 65AY6Li [26], JA-2-3B and JA-3-3Ab [27]; Thermosynechococcaceae: *Thermosynechococcus lividus* PCC 6715 [18], *T. nakabusensis* NK55 [28], *T. sichuanensis* E542 [6], *T*. *taiwanensis* CL-1 [29] and TA-1 [30], *T. vestitus* BP-1 [31], *Thermosynechococcus* sp. M46_R2017_013 and M98_K2018_005 (hereafter M46 and M98) [32]; and Trichocoleusaceae: *Trichothermofontia sichuanensis* B231 [33]. The genome, protein sequences, and genomic annotations of the thermophilic cyanobacteria collected were retrieved from the genomic resources of the NCBI. The genomes with no or incomplete annotations were annotated using the RAST annotation system [34], which were summarized in Supplementary Table 1.

### Identification and classification of TFs

The TF genes were identified in each genome using P2RP (http://www.p2rp.org/, accessed on 20 May 2023) [35]. All the TF genes detected were categorized into families by P2RP based on domain architecture, according to the scheme implemented in the P2CS and P2TF databases [36, 37]. The Pearson coefficient was employed to assess the correlation between the TF gene number and genome size by using the cor.test function in R v3.6.2. Significance levels of 0.05 and 0.01 were applied for the analysis.

### Identification of orthologous proteins

According to the bidirectional best hit (BBH) criterion, orthologous proteins of TF genes were identified in focal taxa using BLASTP. The BLASTP alignments were performed with the following thresholds: identity percentage greater than 40% and query coverage greater than 75% [38].

### Phylogeny

The 16 S rRNA gene sequences were collected for the thermophile collective by retrieval from the NCBI or their extraction from genome sequences. We also included the 16 S rRNA sequences of cyanobacterial references selected according to the phylogenetic relationships based on the literature [39] and the Cyanobacterial Phylogeny and Taxonomy Reference Website. Alignments of 16 S rRNA gene sequences were generated using MAFFT v7.453 [40]. Phylogram of 16 S rRNA gene sequences was inferred using Neighbor-Joining method implemented in MEGA 11.

The similarity clustering was illustrated using an Unweighted Pair Group Method with Algorithmic Mean (UPGMA) based on the AAI (Average Amino acid Identity) matrix estimating all-against-all distances in a collection of focal genomes [41] and visualized using Mega11. Moreover, the 0/1 binary matrix representing the presence or absence of the TF families was used to calculate similarity using Dice’s coefficient [42] for resolving relationships among the thermophiles, and clustering analysis was further performed using UPGMA implemented in NTSYS-pc v.2.10e [43].

## Results and discussion

### Phylogenetic inference of thermophilic cyanobacteria

**Fig. 1:**
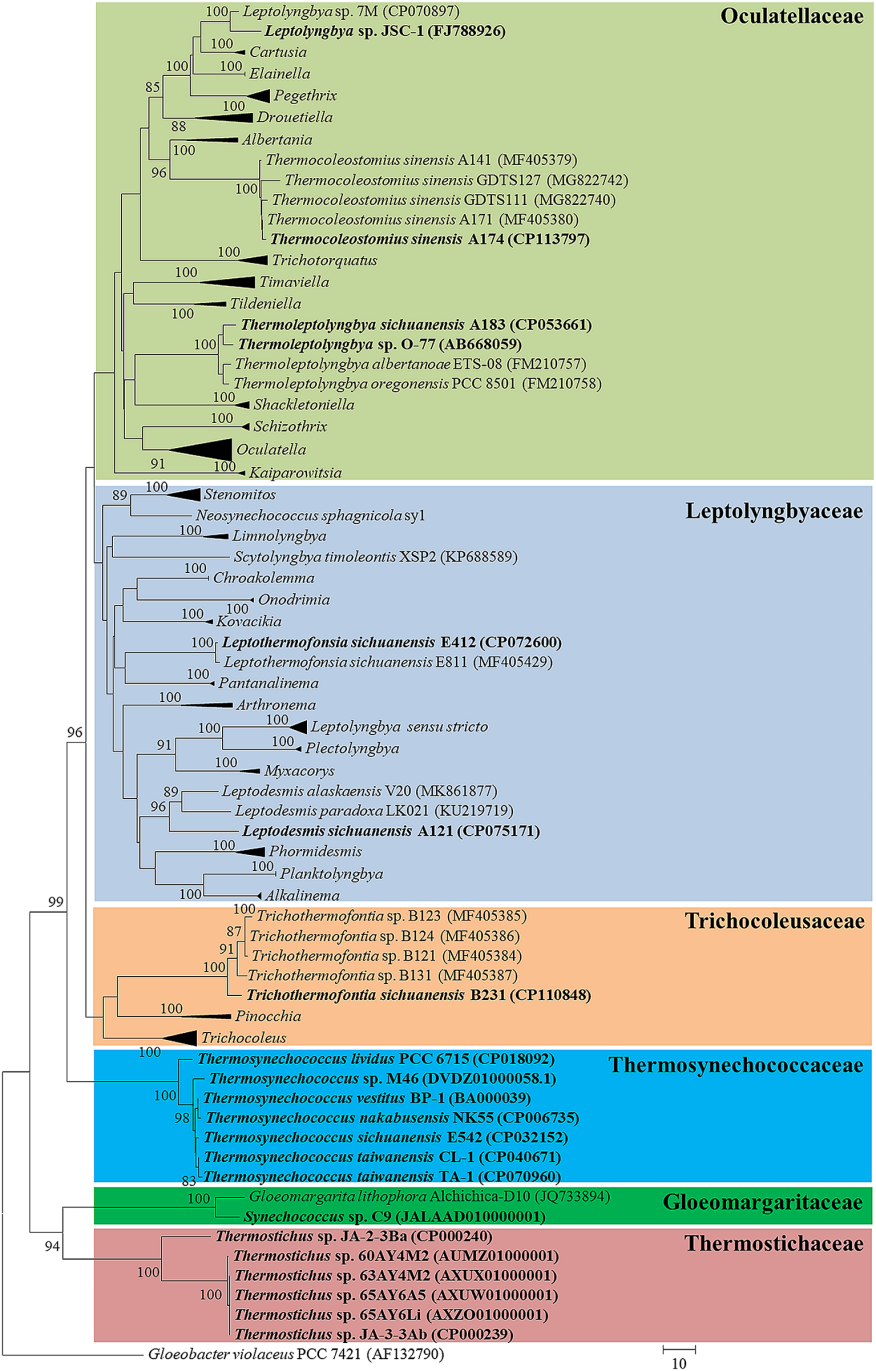
Phylogenetic inference of 16 S rRNA gene sequences representing 128 cyanobacterial strains. Collapsed genera are indicated by black polygons, with a length corresponding to the distance from the most basal sequence to the most diverged sequence of the genus. The thermophilic cyanobacteria are in bold

To better understand the phylogenetic position of the surveyed thermophilic cyanobacteria, 16 S rRNA sequences were employed for phylogenetic inference. The complete phylogram refers to Supplementary Fig. 1. As indicated by Fig. 1, all the thermophilic cyanobacteria were taxonomically assigned to the corresponding genus and family, except for *Leptolyngbya* sp. JSC-1 and *Synechococcus* sp. C9. *Leptolyngbya* sp. JSC-1 appeared to be a novel genus of family Oculatellaceae, rather than a member of genus *Leptolyngbya sensu stricto* within family Leptolyngbyaceae. *Synechococcus* sp. C9 showed an identity of 87.5% to PCC 7942, the type strain of genus *Synechococcus*. Instead, a high similarity (98.2%) was observed between C9 and *Gloeomargarita lithophora* Alchichica-D10 from family Gloeomargaritaceae, suggesting that C9 was not a member of family Synechococcaceae. Both assignments of the two strains are consistent with previous studies that the actual taxonomy of *Leptolyngbya* JSC-1 and *Synechococcus* C9 has not been validated yet [44, 45]. In addition, *Thermosynechococcus* sp. M98 was excluded from phylogenetic analysis due to extremely short sequence of 16 S rRNA. However, whole genome average nucleotide identity (ANI) was calculated for genus and species delineation of this strain. The ANI values between M98 and the other seven *Thermosynechococcus* strains ranged from 83.1 to 92.3%, indicating its allocation as a different species within genus *Thermosynechococcus* according to the suggested values for genus (ANI > 83%) and species (ANI < 96%) delimitation [46].

### TF composition in thermophilic cyanobacteria

**Fig. 2:**
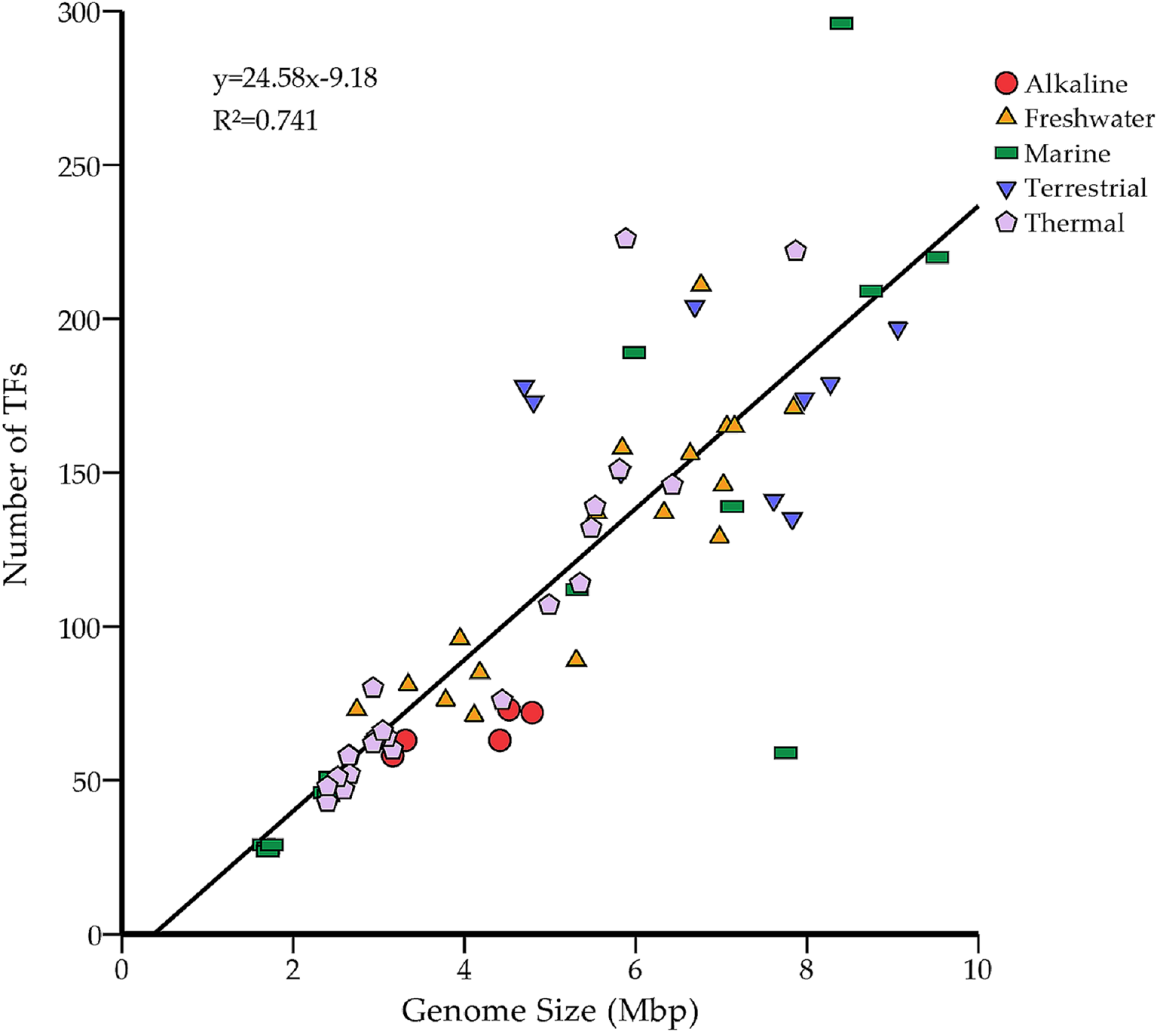
Correlation between transcription factor gene number and cyanobacterial genome size

The predicted TFs from the genomes of 22 thermophilic cyanobacteria were identified and classified, the overall features and detailed results of which were summarized in Table 1 and Supplementary Table 2, respectively. A total of 1,623 TFs were identified in the genomes of these thermophiles. The TFs were classified into four groups based on the P2TF scheme [37], namely transcriptional regulator (TR), one-component system (OCS), response regulator (RR) and sigma factor (SF). TR is the most abundant category of TFs among all the 22 genomes, followed by RR (Table 1). A distinct number of TF genes are exhibited among these genomes (Table 1), tremendously varying from 39 (*Thermosynechococcus* M46) to 210 (*Leptolyngbya* JSC-1). Although the intergenus variation of TF number is evident, different intragenus variations are observed (Table 1). *Thermoleptolyngbya* and *Thermostichus* genomes show a conserved pattern of TF number within the genus, whereas more variation is present among *Thermosynechococcus* genomes. Intriguingly, the number of TF genes appeared to be positively correlated with genome size (Table 1). Thus, we further compiled a genome dataset of 69 cyanobacterial genomes on a larger scale (Supplementary Table 3) to verify the correlation between genome size and TF number. The cyanobacterial genome dataset represented a diverse array of ecological niches, including alkaline, freshwater, marine, terrestrial and thermal niches. The Pearson analysis indicates that the number of TFs is positively associated with genome size (*P* < 0.01) (Fig. 2).

**Table 1:** Summary of putative TF genes in the thermophilic cyanobacteria studied

Species	Genome Size (Mbp)	No. of CDS	Total No. of TFs	No. of TRs	No. of OCSs	No. of RRs	No. of SFs	Percentage of TFs in proteome
*Leptodesmis sichuanensis* A121	5.35	4,809	107	51	18	26	12	2.37%
*Leptolyngbya* sp. JSC-1	7.87	7,423	203	108	40	40	15	2.99%
*Leptothermofonsia sichuanensis* E412	6.43	5,272	120	58	14	33	15	2.77%
*Thermocoleostomius sinensis* A174	5.81	5,532	140	72	24	29	15	2.73%
*Thermoleptolyngbya sichuanensis* A183	5.53	4,402	112	63	19	21	9	3.04%
*Thermoleptolyngbya* sp. O-77	5.48	4,865	119	63	22	23	11	2.71%
*Trichothermofontia sichuanensis* B231	4.44	4,352	68	30	10	17	11	1.75%
*Synechococcus* sp. C9	2.96	3,058	49	27	6	8	8	2.06%
*Thermostichus* sp. 60AY4M2	3.16	2,622	53	22	9	13	9	2.29%
*Thermostichus* sp. 63AY4M2	3.09	2,577	55	23	9	14	9	2.48%
*Thermostichus* sp. 65AY6A5	2.98	2,597	55	24	9	13	9	2.46%
*Thermostichus* sp. 65AY6Li	2.93	2,632	54	23	8	14	9	2.36%
*Thermostichus* sp. JA-2-3Ba	3.05	2,862	57	22	10	14	11	2.31%
*Thermostichus* sp. JA-3-3Ab	2.93	2,760	54	22	9	14	9	2.90%
*Thermosynechococcus lividus* PCC 6715	2.66	2,815	47	21	7	10	9	1.85%
*Thermosynechococcus nakabusensis* NK55	2.52	2,233	47	21	6	13	7	2.37%
*Thermosynechococcus sichuanensis* E542	2.65	2,507	51	25	7	11	8	2.31%
*Thermosynechococcus taiwanensis* CL-1	2.65	2,465	54	23	8	15	8	2.35%
*Thermosynechococcus taiwanensis* TA-1	2.66	2,524	53	23	8	14	8	2.30%
*Thermosynechococcus vestitus* BP-1	2.59	2,475	43	18	6	11	8	1.90%
*Thermosynechococcus* sp. M46	2.40	2,155	39	20	5	7	7	2.00%
*Thermosynechococcus* sp. M98	2.40	2,290	43	21	5	10	7	2.10%

In addition, the proportion of putative TFs in the predicted proteomes ranges from 1.75 to 3.04% (Table 1). Such percentage is in accordance with previous studies regarding prokaryotic organisms, such as *S. elongatus* PCC 7942 (2.7%) [37], but is lower than that of eukaryotic organisms, e.g. unicellular organism *Saccharomyces cerevisiae* (3.5%) [47], multicellular organism *Arabidopsis thaliana* (5.9%) [48]. Moreover, the average proportion in filamentous thermophiles is 2.62%, higher than that (2.27%) in unicellular thermophiles. Previous studies reported that the proportions of TFs in organisms are correlated with the complexity of organisms in light of the fact that TFs play a role in the morphology diversification of organisms [49, 50]. This may explain the higher proportion of TFs in filamentous thermophiles than in unicellular thermophiles. Besides, the subsequent analysis suggests the presence of the TF family unique to specific lineages or species. Thus, it is speculated that the emergence of TF families’ expansion may coincide with the divergence of cyanobacterial lineages [51].

### Comparison of TF families among thermophilic cyanobacteria

**Fig. 3:**
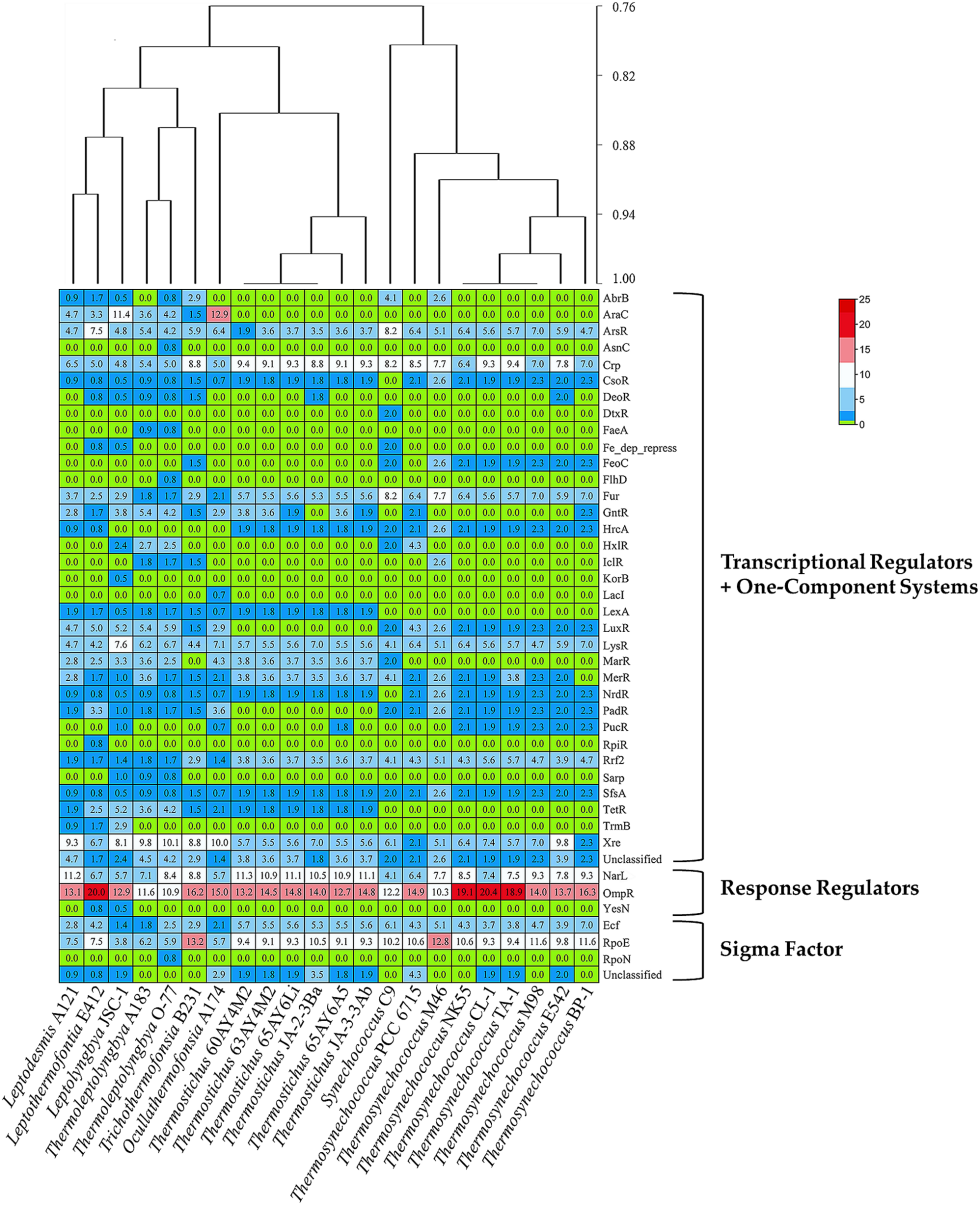
Proportions of TF families in each genome of thermophilic cyanobacteria studied. The plot above is the clustering analysis of thermophilic cyanobacteria based on the presence/absence of TF families. The scale bar indicates the dice coefficient of similarity

TF proteins were sub-categorized into families based on domain organization. A total of 40 TF families as well as unclassified TF families are present in the genomes of the 22 thermophilic cyanobacteria (Fig. 3), including 34 families as TR and OCS, 3 families as RR, and 3 families as SF. Intergenus variations are evident in TF family numbers, while there are extremely limited intragenus variations. The highest TF family number is observed in *Leptolyngbya* JSC-1 (32), followed by *Thermoleptolyngbya* O-77 (31), and *Leptothermofonsia* E412 (30). Overall, filamentous thermophiles show a higher TF family number (28 on average) than unicellular thermophiles (21 on average).

As indicated by Fig. 3 illustrated using TBtools [52], the type of TF family is diverse among the genomes. Eleven TF families are common to all the thermophiles, including ArsR, Crp, Fur, LysR, Rrf2, SfsA, Xre, NarL, OmpR, Ecf, and RpoE family. These TF families account for more than 50% of the TF families in each genome, ranging from 53.81% (*Leptolyngbya* JSC-1) to 81.48% (*Thermosynechococcus* CL-1). The results suggest a conserved pattern of TF families among the surveyed thermophilic cyanobacteria. Particularly, within the genus, the percentages of common TF families are higher than 91.46% and 91.25% in *Thermostichus* and *Thermosynechococcus* genomes, respectively. Such a high proportion in the two genera indicates an extremely conserved genomic core regarding TF families, which is in line with the previous core-genome study of *Thermosynechococcus* genomes [53].

Eight TF families are lineage-specific, namely KorB in *Leptolyngbya* JSC-1, RpiR in *Leptothermofonsia*, LacI in *Thermocoleostomius*, AsnC, FaeA, FlhD and RpoN in *Thermoleptolyngbya*, and DtxR in *Synechococcus*. The AraC family was only found specifically in the surveyed filamentous thermophilic cyanobacteria. Several TF families are absent only in one or two specific lineages, including CsoR and NrdR in *Synechococcus*, LuxR and PadR in *Thermostichus*, and LexA, MarR and TetR in *Synechococcus* and *Thermosynechococcus*.

The lineage-specific TF families may reveal the evolutionary history of these thermophiles. Therefore, cluster analysis was performed using a binary matrix representing the presence or absence of the TF families to infer the relationships among the thermophiles. As shown in Fig. 3, each lineage is well separated from the other due to the presence of TF families specific to the lineage. The clustering of thermophiles is consistent with genus-level taxonomic assignment of these lineages in the phylogram of 16 S rRNA (Fig. 1). At the family level, discrepancy is noticed. JSC-1 from family Oculatellaceae appeared to be closer to the two genera from family Leptolyngbyaceae, while A174 was located in a separate branch, rather than with other genera from family Oculatellaceae. The incompatible topologies indicate that the evolution of TF families is partially inconsistent with phylogenetic relationship of these thermophiles. In addition, sub-clusters are also noticed within genus *Thermostichus* and *Thermosynechococcus*, suggesting the divergence of TF families in the two genera during their evolutionary process.

### Putative functions of common and specific TF families among thermophilic cyanobacteria

The 11 common TF families among thermophilic cyanobacteria showed diverse putative functions and may regulate the transcription of key genes involved in the acclimation responses to environmental changes. The two common response regulators, OmpR and NarL family, constituted the overwhelming majority of the putative RRs identified in each genome. The OmpR family may function as a key regulator to mediate a wide range of biological functions related to osmolarity, phosphate assimilation, antibiotic resistance, virulence and toxicity [54], whereas the NarL family was documented to control the expression of genes related to nitrogen fixation, sugar phosphate transport, nitrate and nitrite metabolism, quorum sensing, and osmotic stress [55]. Similarly, the vast majority of the putative sigma factors identified in each genome were classified into the following two families: Ecf and RpoE family, both of which exhibit the extracytoplasmic function. The RpoE family can trigger the expression of genes protecting against photooxidative stress, and may partially overlap with the heat-shock response [56]. The Ecf family may be involved in DNA repair response, cell wall stress, and amino acid starvation [57, 58].

The remaining seven common TF families were grouped into one-component systems and transcriptional regulators. The Xre family may play important roles in pathogenicity and virulence mechanisms, e.g. biofilm formation, quorum sensing, and homeostasis [59, 60]. The Crp family adjusts global gene expression in response to the C-to-N balance in the cells, and the LysR family extensively facilitates the acclimation of cells to oxygenic phototrophy [61]. The metalloregulator ArsR family is involved in stress response to metal ions in cyanobacteria [62]. Another metal regulon, the Fur family, controls genes for iron/zinc acquisition or genes involved in oxidative stress [63]. Members of the Rrf2 family regulate gene expression to enable adaptation, maintenance of homeostasis and cell protection [64]. Moreover, all the genomes of thermophilic cyanobacteria contain a SfsA TF that is known to be involved in sugar fermentation [65].

In addition to the common TF families, lineage-specific TF families might be more important for themselves to perform unique regulation of gene expressions. The KorB family unique in *Leptolyngbya* may control the replication [66]. The RpiR may be a transcription activator responsible for sugar catabolism in *Leptothermofonsia* [67]. The LacI family in *Thermocoleostomius* may sense sugar effectors and regulate carbohydrate utilization genes [68]. The TF families unique in *Thermoleptolyngbya* may be involved in various biological processes, e.g. amino acid metabolism and transport by the AsnC family [69], DNA methylation by the FaeA family [70], and nitrogen and carbon metabolism by RpoN family [71]. Although the FlhD was identified in *Thermoleptolyngbya*, FlhC was absent from the genome. Given the fact that the *flhDC* operon is essential for the transcription of all the genes in the flagellar cascade [72], it is unclear that the FlhD in *Thermoleptolyngbya* act as a global regulator involved in cellular processes or not. *Synechococcus* possessed a unique metalloregulator, the DtxR family, which may regulate the expression of genes involved in metal homeostasis in the cell, particularly genes for manganese uptake transporters [73]. The AraC family may participate in the control of genes involved in important biological processes such as carbon source utilization, morphological differentiation, secondary metabolism, pathogenesis and stress responses [74].

The CsoR family is widely distributed and regulates the regulons involved in detoxification in response to extreme copper stress [75]. The absence of CsoR in *Synechococcus* C9 suggests that an alternative mechanism may be utilized for copper resistance. In addition, *Synechococcus* C9 excluded another widely conserved regulator, NrdR, of Ribonucleotide reductase genes [76]. LuxR and PadR are widespread and functional diverse transcription factors [77, 78], which are missing in *Thermostichus*. The LexA, MarR and TetR are absent in *Synechococcus* and *Thermosynechococcus* genomes. LexA regulates gene expression in response to environmental changes (e.g. salt stress) [79], MarR is critical for bacterial cells to respond to chemical signals and to convert such signals into changes in gene activity [80], and TetR regulates various essential processes (e.g. metabolism, biofilm formation and efflux gene expression) [81]. The missing conserved TF families might have no lethal impact on these thermophiles to survive in hostile thermal niches. Conversely, the corresponding functions of regulation may be compensated by other TFs, since most TFs are global multi-target regulators. Moreover, the presence or absence of conserved TF families among these thermophilic cyanobacteria could be explained either by the loss of these families during evolutionary history or by the acquisition of these families by horizontal gene transfer.

### Orthologous TFs in *thermostichus* and *thermosynechococcus*

Only *Thermostichus* and *Thermosynechococcus* strains were included for the identification of orthologous TF proteins, since the other genera studied have only one or two strains sequenced for whole genomes. The orthologous TF proteins shared by the *Thermostichus* or *Thermosynechococcus* **s**trains were summarized in Supplementary Table 4. As for the six *Thermostichus* genomes, 300 (91.5%) out of 328 putative TF genes were common to all the genomes, suggesting a quite conserved core set of TF genes in these *Thermostichus* strains. Regarding the eight *Thermosynechococcus* genomes, 256 (67.9%) out of 377 putative TCS genes were common to all the genomes (Supplementary Table 4). Compared to the core set in *Thermostichus* strains, relatively less conserved TF genes was shown by the *Thermosynechococcus* genomes.

### Accessory TFs in *thermostichus* and *thermosynechococcus*

**Fig. 4:**
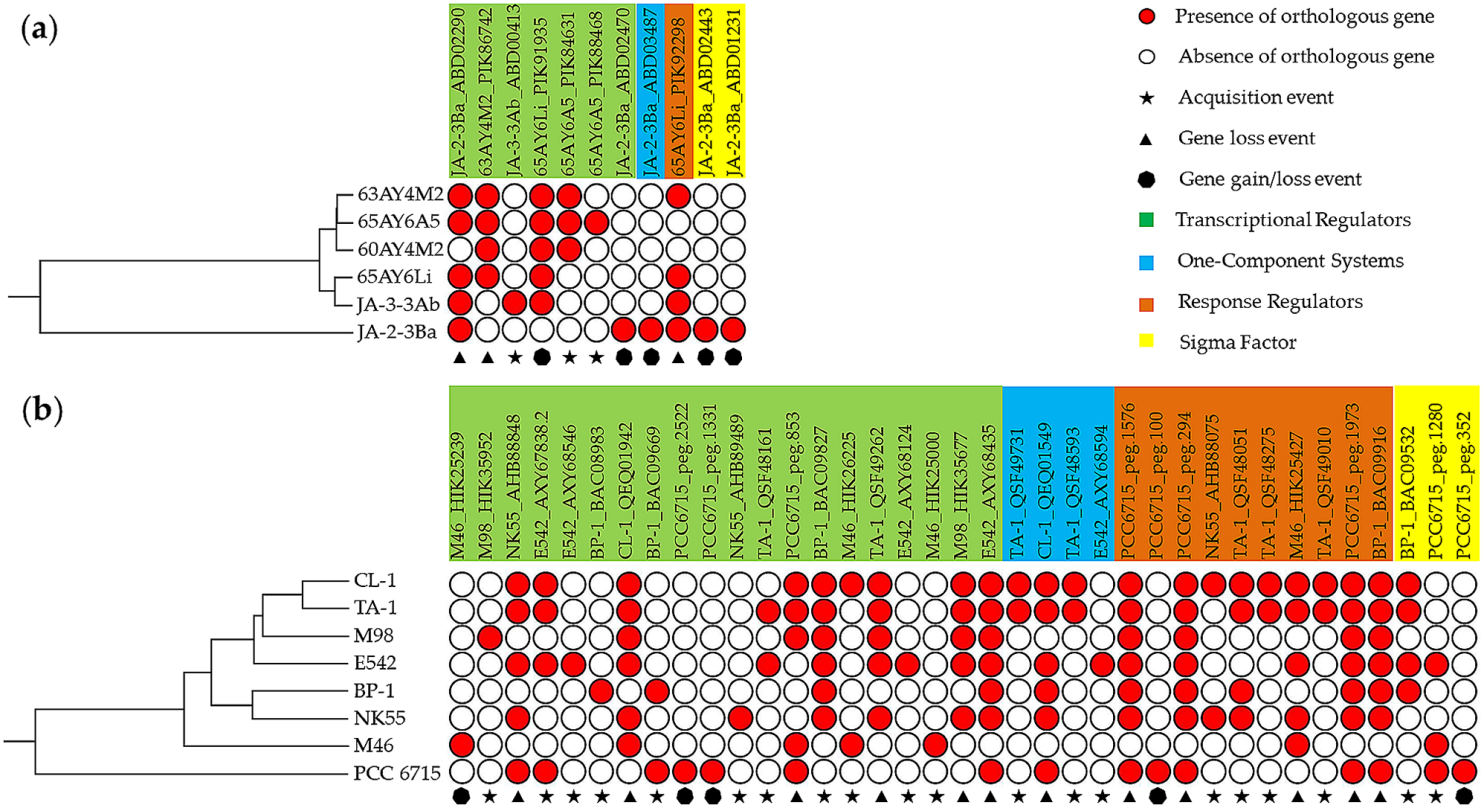
Occurrence of accessory TF genes in *Thermostichus* (**a**) and *Thermosynechococcus* strains (**b**). The UPGMA trees on the left were built based on AAI distances of focal genomes. The TFs were named by the strain name plus accession number and only one ortholog was shown. The full dataset was summarized in Supplementary Table 4

Apart from the core set of TF genes, accessory TF genes might be biologically more important due to their contribution to the genome plasticity. In these *Thermostichus* genomes, 28 TF genes were identified as accessory (Supplementary Table 4). Among them, six were strain-specific TFs, four of which were affiliated with JA-2-3Ba (Fig. 4a). A total of 121 TF genes were found to be accessory in these *Thermosynechococcus* genomes (Supplementary Table 4). Only 12 TF genes were strain-specific (Fig. 4b), contributed by PCC 6715 (four genes), E542 (three genes), M46 (two genes), and NK55, BP-1 and M98 (one gene each).

In addition, comparative and evolutionary genomic analyses were conducted to infer the origin of accessory TF in *Thermostichus* and *Thermosynechococcus* strains. Among the 28 accessory TF genes in the *Thermostichus* genomes, three gene loss events and three gene acquisition events were putatively identified, while five gene gain/loss events may occur during the evolutionary process (Fig. 4a). The gene loss events occurred either recently in a single *Thermostichus* strain or a common ancestor of *Thermostichus* strains, both indicating the possible recent loss events. Among the three gene acquisition events, two TFs (JA-3-3Ab_ABD00413 and 65AY6A5_PIK88468) may be independently acquired by the two strains; and the three TFs (65AY6A5_PIK84631, 63AY4M2_PIK86529 and 60AY4M2_PIK95596) may be acquired by the common ancestor of the three strains.

Regarding the 121 accessory TF genes in the *Thermosynechococcus* genomes, 12 gene loss events and 21 acquisition events were putatively identified, whereas four gene gain/loss events were observed (Fig. 4b). Numerous gene loss events and acquisition events were associated with PCC 6715, which might contribute to the divergence indicated by the phylogeny of this species among the *Thermosynechococcus* strains (Fig. 4b). The gene loss events were complex among the *Thermosynechococcus* strains, which occurred recently in a single strain, a common ancestor of strains, or independently in two or three strains. Similarly, acquisition events were also complicated, which happened only in a single strain, in several strains, in a clade, and in a common ancestor.

Conclusively, HGT events may be involved in the evolutionary history of TF genes in *Thermostichus* and *Thermosynechococcus* strains. The results regarding *Thermosynechococcus* strains were in accordance with previous reports that numerous putative genes horizontally transferred from other bacteria have been actively acquired by *Thermosynechococcus* species, conferring the acclimation of them to stressful niches in hot springs [29, 82]. However, fewer HGT events were found in *Thermostichus* strains, which were inhabited in niches that were near 73^o^C [83]. This could be explained by a tentative hypothesis that extremely hot spring environments may provide more limited opportunity for lateral gene transfer, which in turn could lead to less opportunity for lateral gene transfer [53]. Taken together, the results indicated genome plasticity of TF genes may be used for coping with unique challenges that strains of the two genera faced in hostile habitats.

### Comparative analysis of TFs between thermophilic and mesophilic cyanobacteria

We further compared the TFs between the surveyed thermophilic cyanobacteria and mesophilic cyanobacteria. According to the family-level allocation in the 16 S rRNA phylogram (Supplementary Fig. 1), one reference genome of mesophilic cyanobacteria was selected from each family based on the genome quality, ecological niches and/or temperature characteristics. The TFs of each genus were compared to the TFs of corresponding reference genome. *Synechococcus* PCC 7942 was used as the reference since there’s no other genus within family Thermosynechococcaceae and Thermostichaceae. The identified genus-specific and strain-specific TFs were further compared to TFs of *Synechocystis* PCC 6803 for function prediction.

Within family Oculatellaceae, *Elainella* E1 was selected as reference, which was isolated from ephemeral waterbody (26.7^o^C, pH 6.06) in the forest, Cat Tien National Park, Vietnam [84]. Numerous TFs of the two *Thermoleptolyngbya* strains, *Thermocoleostomius* A174, and *Leptolyngbya* JSC-1 were identified to be unique to the TFs of *Elainella* E1, respectively (Supplementary Table 4). The identified unique TFs belong to diverse types (Supplementary Table 4), among which AraC family appears to be more abundant in these thermophiles. Several AraC TFs are orthologous to the TFs (*sll1205*, *sll1408* and *sll1489*) of *Synechocystis* PCC 6803 (Supplementary Table 5), which may function as iron stress regulon in these thermophiles [85]. In addition, *Thermoleptolyngbya* O-77 has a homolog of *sll0184* that is involved in the acclimation to low inorganic carbon during the prolonged high temperature [86], while *Leptolyngbya* JSC-1 possesses a homolog of *sll1594*, a repressor of the genes encoding components of Ci transporters, such as the NDH complex and the high-affinity sodium/bicarbonate symporter *SbtA*, under high carbon conditions [87]. Both TFs may confer the thermophilic cyanobacteria with more alternative strategies to survive in environments with significant CO_2_ fluctuation, particularly in hot springs [18]. Moreover, many TFs from these thermophiles are also identified to be homologs of the TFs in *Synechocystis* PCC 6803 that are involved in regulation in responses to environmental changes, such as nitrogen, nitrate, metal and phosphate (Supplementary Table 5).

Around 38% of the TFs in *Leptodesmis* A121 and *Leptothermofonsia* E412 (Supplementary Table 4) are separately unique to the TFs of *Leptolyngbya* dg5, a freshwater cyanobacterium. Two unique TFs of the two thermophiles are annotated as regulon to metals (Supplementary Table 5). Unfortunately, no putative functions were predicted for the other unique TFs.

*Trichothermofontia* B231 was compared to *Trichocoleus* FACHB-46, a terrestrial cyanobacterium [13]. Around 70% of the TFs in *Trichothermofontia* B231 are orthologous to that of FACHB-46 (Supplementary Table 4), may suggesting a similar TF composition.

Compared to *Synechococcus* PCC 7942, homologs are found to account for around 70% of the TFs in each *Thermosynechococcus* genome (Supplementary Table 4). Four *Thermosynechococcus*-specific TFs are homologs of *sll0176*, *sll1371*, *slr1783* and *slr1909* (Supplementary Table 5), may participating metal responses, twitching motility, acid tolerance and other transcription regulation [88–91]. The strain-specific TF (PCC6715.100) from PCC 6715 (Supplementary Table 5) may be involved in cadmium tolerance and metal homeostasis [92].

Within family Gloeomargaritaceae, *G. lithophora* is to date the only described genus, Alchichica-D10 as the type strain [93].The mesophilic strain grew within a temperature range of 15–30 °C, whereas thermophilic C9 can grow at up to 55 °C [25]. A total of 38 orthologous TFs are shared by the two strains and the 11 TFs unique to C9 are all TR, representing seven TF types (Supplementary Table 4). Among them, the TF (C9.469), a homolog of *sll5035*, may be involved in arsenic resistance of C9 and obtained via horizontal gene transfer [94]. In addition, the TF (C9.1780) is homologous to *sll7009*, a negative regulator specific for the CRISPR1 subtype I-D system in *Synechocystis* PCC 6803 [95]. And this TF has no homologs or shares similar functions of other TFs in the other surveyed thermophiles. This result indicates that some regulations are limitedly shared by thermophilic and mesophilic cyanobacteria. More importantly, a comprehensive genomic comparison is essential in future to elucidate the thermal difference between the two strains.

Unlike *Thermosynechococcus*, approximately half of TFs in each *Thermostichus* genome are orthologous to that of *Synechococcus* PCC 7942 (Supplementary Table 4). The *Thermostichus*-specific TFs are homologs of *sll1371* and *slr1909* (Supplementary Table 5), may functioning for twitching motility and acid tolerance [89, 91]. The strain-specific TF (JA-2-3Ba_ABD02443.1), a homolog of *sll1689*, may facilitate this strain to cope with nitrogen starvation [96].

Overall, the comparisons suggest a different TF composition between the surveyed thermophiles and corresponding mesophiles. Furthermore, the identified TFs of thermophiles that are distinct from that of mesophiles are putatively involved in various biological regulations, mainly as responses to ambient changes. Those potential regulatory systems may benefit the thermophiles to survive in hot springs, and may represent the characteristic molecular signatures in these thermophiles and should be therefore experimentally elucidated in the near future.

## Conclusions

Herein, a thorough investigation and comparative analysis was carried out regarding the composition and abundance of TFs in 22 thermophilic cyanobacteria. The results suggested a fascinating diversity of the TFs among these thermophiles. The abundance and type of TF genes were diversified in these genomes. The identified TFs are speculated to play various roles in biological regulations. Further comparative and evolutionary genomic analyses revealed that HGT may be associated with the genomic plasticity of TF genes in *Thermostichus* and *Thermosynechococcus* strains. Comparative analyses also indicated different pattern of TF composition between thermophiles and corresponding mesophilic reference cyanobacteria. Moreover, the identified unique TFs of thermophiles are putatively involved in various biological regulations, mainly as responses to ambient changes, may facilitating the thermophiles to survive in hot springs. Conclusively, the obtained findings provided insights into the TFs of thermophilic cyanobacteria and fundamental knowledge for further research regarding thermophilic cyanobacteria with a broad potential for transcription regulations in response to environmental fluctuations.

### Electronic supplementary material

Below is the link to the electronic supplementary material.

**Supplementary Material 1**: Genome annotations of thermophilic cyanobacteria

**Supplementary Material 2**: Predicted TF genes in the genome of cyanobacteria studied

**Supplementary Material 3**: Habitat and number of TF genes of cyanobacteria studied

**Supplementary Material 4**: Phylogenetic inference of 16S rRNA gene sequences representing 128 cyanobacterial strains

**Supplementary Material 5**: Ortholog table of TFs in strains from each family

**Supplementary Material 6**: Blastp results between TFs of thermophilic cyanobacteria and TFs of *Synechocystis* PCC 6803

## Data Availability

The data presented in this study are openly available in the National Center for Biotechnology Information (https://www.ncbi.nlm.nih.gov/genome/).
