# Supplementary figures and images for: Genome-scale identification and comparative analysis of transcription factors in thermophilic cyanobacteria
Source: BMC Genomics. 2024 Jan 9;25:44. doi: 10.1186/s12864-024-09969-7 (PMC10775510; doi:10.1186/s12864-024-09969-7)

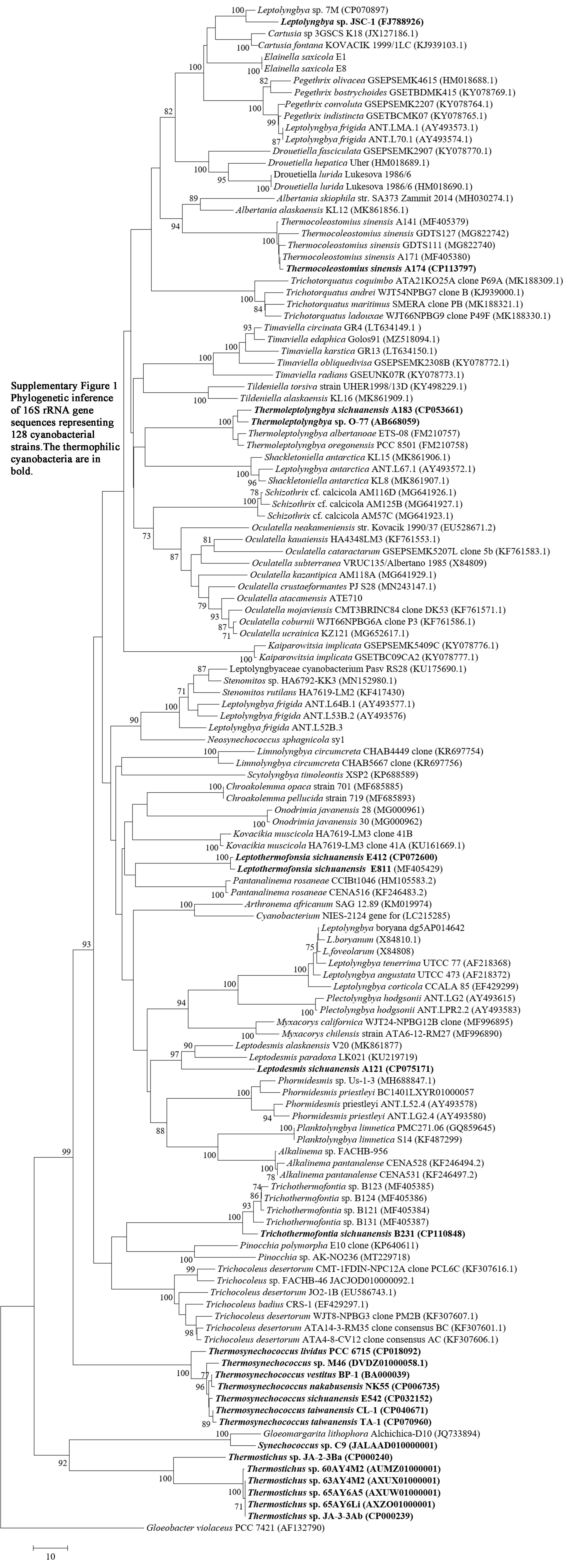

Supplement: Supplementary file 4 — Supplementary Material 4: Phylogenetic inference of 16S rRNA gene sequences representing 128 cyanobacterial strains [file 12864_2024_9969_MOESM4_ESM.tif]
